# Hypertonic Saline Induces Host Protective Immune Responses against *Brucella abortus* Infection in Mice

**DOI:** 10.4014/jmb.2407.07040

**Published:** 2024-09-11

**Authors:** Tran Xuan Ngoc Huy, Trang Thi Nguyen, Said Abdi Salad, Ched Nicole Turbela Aguilar, Alisha Wehdnesday Bernardo Reyes, Lauren Togonon Arayan, WonGi Min, Hu Jang Lee, Huynh Tan Hop, Suk Kim

**Affiliations:** 1 Institute of Applied Sciences, HUTECH University, 475A Dien Bien Phu St., Ward 25, Binh Thanh District, Ho Chi Minh City, Viet Nam; 2 Institute of Animal Medicine, College of Veterinary Medicine, Gyeongsang National University, Jinju, 52828, Republic of Korea; 3 Department of Veterinary Paraclinical Sciences, College of Veterinary Medicine, University of the Philippines Los Baños, College, Laguna, 4031, Philippines; 4 The Jackson Laboratory, Bar Harbor, ME, USA; 5 University Center for Bioscience and Biotechnology, National Cheng Kung University, Tainan, Taiwan

**Keywords:** *Brucella abortus*, hypertonic saline, immune response, cytokines, transcription factor

## Abstract

Hypertonic saline (HTS) resuscitation can enhance immune responses against various pathogens, however, the effect of HTS on brucellosis is yet to be defined. In this study, we found that HTS inhibited *Brucella* infection in mice by augmenting Th1 immunity. HTS treatment enhanced the serum cytokines production and the expression of nitric oxide synthase (NOS2) and nuclear factor kappa B (NF-ĸB) p50 and p65, crucial anti-*Brucella* effectors in splenocytes. In addition, HTS treatment also inhibited the phosphorylation of MAPK signaling, accompanied by the down-regulation of the autophagy marker LC3B-II. Due to directing an appropriate immune response, HTS treatment substantially decreased bacterial burden in spleen and liver tissues. In summary, corroborating previous studies showing the antimicrobial effects of HTS, our findings indicate that HTS treatment triggers a protective immune response against *Brucella* infection. Additionally, these results provide promising evidence of the immunomodulatory role of HTS in controlling bacterial infections.

## Introduction

*Brucella* (*B.*) species are Gram-negative, facultative intracellular bacteria. They are the causative agents of brucellosis in animals and humans. To date, twelve species of *Brucella* have infected various hosts including *B. abortus* (cattle and buffaloes), *B. melitensis* (sheep, goats and camels), *B. canis* (dogs), *B. ovis* (sheep), *B. inopinata* (human), *B. suis* (pigs, hares, reindeers and wild boars), *B. microti* (common vole), *B. neotomae* (desert woodrats), *B. papionis* (baboons), *B. vulpis* (foxes), *B. pinnipedialis* (seals, sea lions and walruses), *B. ceti* (whales, porpoises and dolphins). *Brucella* infection has spread widely among terrestrial and marine mammals [1]. Among them, *B. abortus*, *B. melitensis* and *B. suis* are the most common causes of infection in humans with approximately 1.6 to 2.1 million new cases annually, putting approximately 3.5 billion people at risk for brucellosis globally [2, 3]. However, there are currently no adequate licensed vaccines for preventing human brucellosis while the live attenuated vaccines including *B. abortus* S19, *B. abortus* RB51 and *B. melitensis* Rev.1 have been mostly used to prevent animal brucellosis [4]. In the context of brucellosis treatment, criteria such as a decrease in symptomatic time, relapse, complications, mortality or chronicity are proposed gold standards. Based on these criteria, the uses of antimicrobial drugs, surgical treatments, traditional medicine, or nanotechnology therapy yield protective efficiency but have been controversial [3]. Therefore, alternative therapies for treating or preventing brucellosis related to immunomodulation and immunometabolism that target host and pathogen interactions have recently been investigated as an emerging field [5].

Hypertonic saline (HTS) resuscitation or hypertonic saline infusion affects a wide range of physiological and immune responses. In particular, HTS treatment mediates hemodynamics, efficient plasma volume expansion, electrolyte changes as well as functions of immune cells such as neutrophil granulocytes, mononuclear phagocyte systems, natural killer cells, lymphocytes, and complement systems [6-8]. A study conducted by Coimbra *et al*. [9] demonstrated a protective efficiency of HTS against mortality and bacteremia in sepsis after hemorrhage in a BALB/c mouse model. Interestingly, HTS was demonstrated to affect host inflammatory responses through regulation of cytokines/chemokines, signaling pathways/transcription factors expression. HTS is considered an anti-inflammatory agent, effectively reducing pro-inflammatory responses in the primary human small airway lung epithelial cells. In addition, the anti-inflammatory effect of HTS was observed in an acute pancreatitis rat model, where the expression of Cox2, nitric oxide (NO), and inflammatory cytokines was inhibited in HTS-treated rats [10, 11]. On the contrary, using 3% of NaCl induced inflammatory responses in human THP-1 macrophage cells benefits patients suffering from chronic airway disease [12]. Notably, another study where mice were subjected to sepsis by infection with a sublethal dose of live *Escherichia* (*E.*) *coli*, followed by HTS treatment, led to the enhancement of host protective responses to eliminate bacterial infection [13, 14]. In addition, a high salt diet was proven to protect mice from the lethal vesicle stomatitis virus infection, and HTS inhibited the growth of *Pseudomonas aeruginosa* isolated from cystic fibrosis patients [15, 16]. With potential effects on host immunity against bacterial infection and trauma, we hypothesize that HTS can trigger immunomodulatory responses against *B. abortus* infection, which has not yet been elucidated. Particularly, in this study, BALB/c mice were subjected to HTS treatment during *Brucella* infection. Afterward, immunological properties stimulated by HTS including pro-inflammatory, anti-inflammatory cytokines production, cell signal transduction and autophagy expression were evaluated.

## Materials and Methods

### Animals and Bacterial Growth Condition

Female BALB/c mice, eight weeks old (Samtako, Inc., Republic of Korea), were randomly allocated into four experimental groups with six mice per group. All mice were acclimatized for one week before bacterial infection. The animal experiment protocol in this study was approved by the Animal Ethical Committee of Chonbuk National University (Authorization Number JBNU-2022-017-001).

The smooth, virulent, wild-type *B. abortus* 544 biovar 1 strain (ATCC 23448) was cultured in *Brucella* broth (BBL BD, USA) at 37°C with vigorous shaking for two days until stationary phase at a biosafety laboratory level 3 (BSL3) containment facility. In addition, viable bacteria were maintained and measured by streaking or plating serial dilutions on *Brucella* broth containing 1.5% agar (VWR, USA), respectively.

### Animal Experiments and Protection Assay

After one week of acclimation, two groups were not infected, and another two groups were intraperitoneally infected with *B. abortus* 544 at a concentration of 5 × 10^5^ colony forming unit (CFU)/mouse in a total volume of 100 μl in PBS. After infection, mice were received 100 μl HTS (200 mM NaCl) or PBS orally by gavage and daily for 14 days. At day 14 post-infection, peripheral blood samples were collected via tail vein to obtain sera and then all mice were sacrificed. Spleen and liver samples were collected, and their total weights were measured to determine splenomegaly and hepatomegaly. To evaluate the effect of HTS treatment on protective efficiency against *Brucella* infection, a 0.05 g part of the spleen or liver was aseptically sliced and homogenized in 1 ml PBS and subjected to 10-fold dilution using PBS. Finally, 50 μl of the dilution was plated onto Brucella agar, and the base-10 logarithm of the number of CFU of each sample was calculated.

### Serum Cytokines/Chemokines Level Measurement

Sera were collected from peripheral blood by centrifugation at 2,000 ×*g* at 4°C for 10 min. The concentration of IFN-γ, IL-10, TNF-α, IL-6 cytokines and MCP-1 chemokine were measured by Cytometric Bead Array (CBA Mouse inflammation kit, BD Biosciences, USA). Data were acquired and analyzed using BD FACSVerse flow cytometry. The production of IL-2 and IL-4 cytokines were determined by sandwich ELISAs (Abcam, USA) following the manufacturer’s instructions.

### Total RNA Extraction from Spleen and cDNA Synthesis

At 14 days post-infection, spleens were collected and the total RNA in spleen was extracted using Riboex reagent (Geneall, Korea) and RNeasy Mini kit (Qiagen, Germany). All the procedures were performed according to the manufacturer’s instructions. In addition, the genomic DNA contamination was removed by RNase-free DNase (Qiagen). The concentration of purified RNA was measured using a Nanodrop UV/VIS spectrophotometer (Optizen, Republic of Korea). A 1 μg of purified RNA was reverse transcripted into complementary DNA (cDNA) using a Quantitech Reverse Transcription Kit (Qiagen) for all samples. The synthesized cDNA was used as the template for the quantitation of gene expression in splenic cells.

### Quantitative Real-Time PCR (qRT-PCR)

The expression of genes related to mouse immune responses was assessed through qRT-PCR. Briefly, a qRT-PCR reaction mixture contained a total volume of 20 μl including cDNA template, distilled water, primer pairs and SYBR Green master mix (Promega, USA). qRT-PCR was performed on CFX96 Touch Real-Time PCR Detection System (Bio-Rad, USA) with the following thermal cycling conditions: 95°C for 10 min, 39 cycles with 95°C for 15 s and 60°C for 1 min. The melting curve analysis was performed from 65°C to 95°C with an increment of 1°C each 5 s to confirm the amplification efficiency of each primer pair for their specific gene. The acquired results were analyzed using Bio-Rad CFX Manager software, version 3.1. The relative fold change of the mRNA level was quantified using ΔΔC_t_ method with β-acin used as a reference gene. The list of all primer pairs are given in Table 1.

### Total Protein Extraction from Spleen Samples

The spleen samples were lysed in Radioimmunoprecipitation assay (RIPA) lysis buffer (Santa Cruz, USA) following the manufacturer’s protocol. After centrifugation at 13,000 ×*g* for 15 min at 4°C, the supernatant containing the total proteins was collected. After that, the total protein concentration was measured using the Bradford method (Bio-Rad). All samples were subjected to sodium dodecyl sulfate-polyacrylamide gel electrophoresis (SDS-PAGE) and western blotting assay.

### Immunoblot Analysis

All samples containing equal amounts of proteins were denatured and reduced by boiling in 2x SDS buffer (Bio-Rad) for 5 min. After that, all samples were separated by SDS-PAGE. The proteins were transferred onto Immobilon-P membranes (Millipore, USA) using 1x transfer buffer (25 mM Tris, 192 mM glycine and 20%methanol). The membranes were then blocked with blocking buffer (5% skim milk in phosphate-buffered saline containing 0.1% Tween 20, PBS-T) at room temperature (RT) for 30 min and probed with the primary antibodies to detect phospho-p38, phospho-ERK1/2, phospho-JNK, phospho-NF-ĸB-p50 and p60, LC3B-I, LC3B-II, iNOS and IL-1β overnight at 4°C. The membranes were washed three times with PBS-T and incubated with horseradish peroxidase-conjugated secondary antibody for 1 h at RT, followed by three times washing with PBS-T. Finally, the signal was detected using ECL reagents (Atto Corp., Japan). The immunoreactive bands were visualized by the Molecular Imager ChemiDoc XRS system machine (Bio-Rad Laboratories, USA). β-actin was used as a housekeeping protein. All primary and secondary antibodies were purchased from Cell Signaling (USA) and diluted with the appropriate ratio in blocking buffer.

### Statistical Analysis

The results for each experiment are expressed as the mean ± standard deviation (SD) from at least two independent experiments with at least two technical replicates in each experiment unless otherwise defined in the figure legends. Statistical analyses were performed with one-way or two-way ANOVA followed by multiple comparisons among groups in Prism (GraphPad Software;*, *p* < 0.05; **, *p* < 0.01; ***, *p* < 0.001; ****, *p* < 0.0001; ns, not significant).

## Results

### Profile of Pro-Inflammatory and Anti-Inflammatory Cytokines/Chemokine Secretion in Serum

**Fig. 1:**
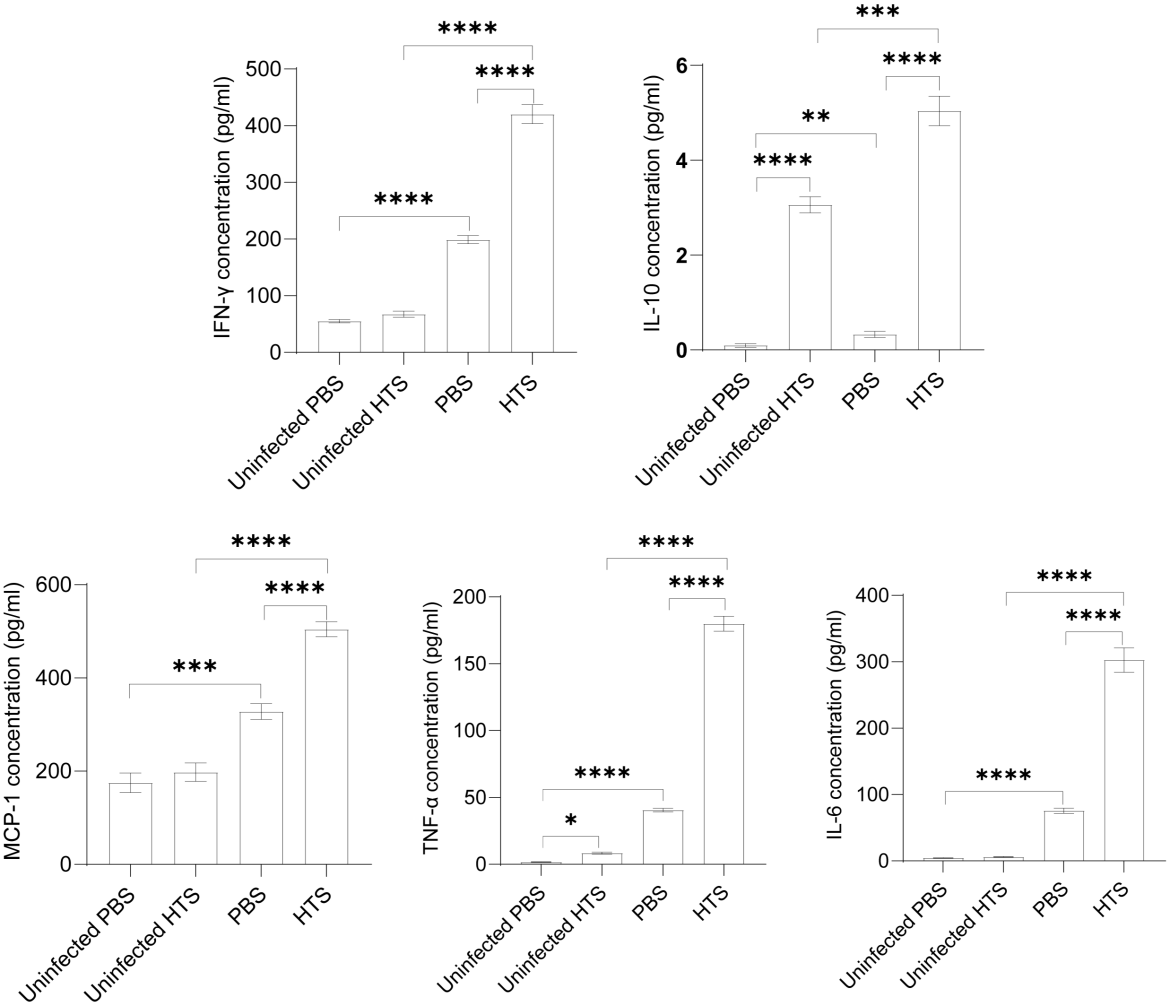
Cytokine concentrations in the sera of mice treated with HTS. Sera from peripheral blood samples were collected from the tail vein on the 14^th^ day post-infection. The concentrations of IFN-γ, IL-10, MCP-1, TNF-α and IL-6 were analyzed using a cytometric bead array. The data are represented as the mean ± SD of the mean of each group of six sera samples. Asterisks indicate statistically significant differences (*, *p* < 0.05; **, *p* < 0.01; ***, *p* < 0.001; ****, *p* < 0.0001).

Host pro- and anti-inflammatory responses were triggered upon *Brucella* infection [17]. The secretion of pro-inflammatory and anti-inflammatory cytokines/chemokines in sera modulates host immunity against *Brucella* infection. In the uninfected groups, HTS treatment significantly increased the production of TNF-α and IL-10, while there were no differences in the secretion of IFN-γ, MCP-1 and IL-6 between treated and untreated groups. Interestingly, in the infected groups, the results showed a marked enhancement of cytokines IFN-γ, IL-10, TNF-α and IL-6 and chemokine MCP-1 production in the HTS-treated mice compared to untreated mice. These results suggested that HTS treatment significantly activated both pro- and anti-inflammatory responses in the host during *Brucella* infection (Fig. 1).

### HTS Treatment Substantially Increased Host Inflammation

**Fig. 2:**
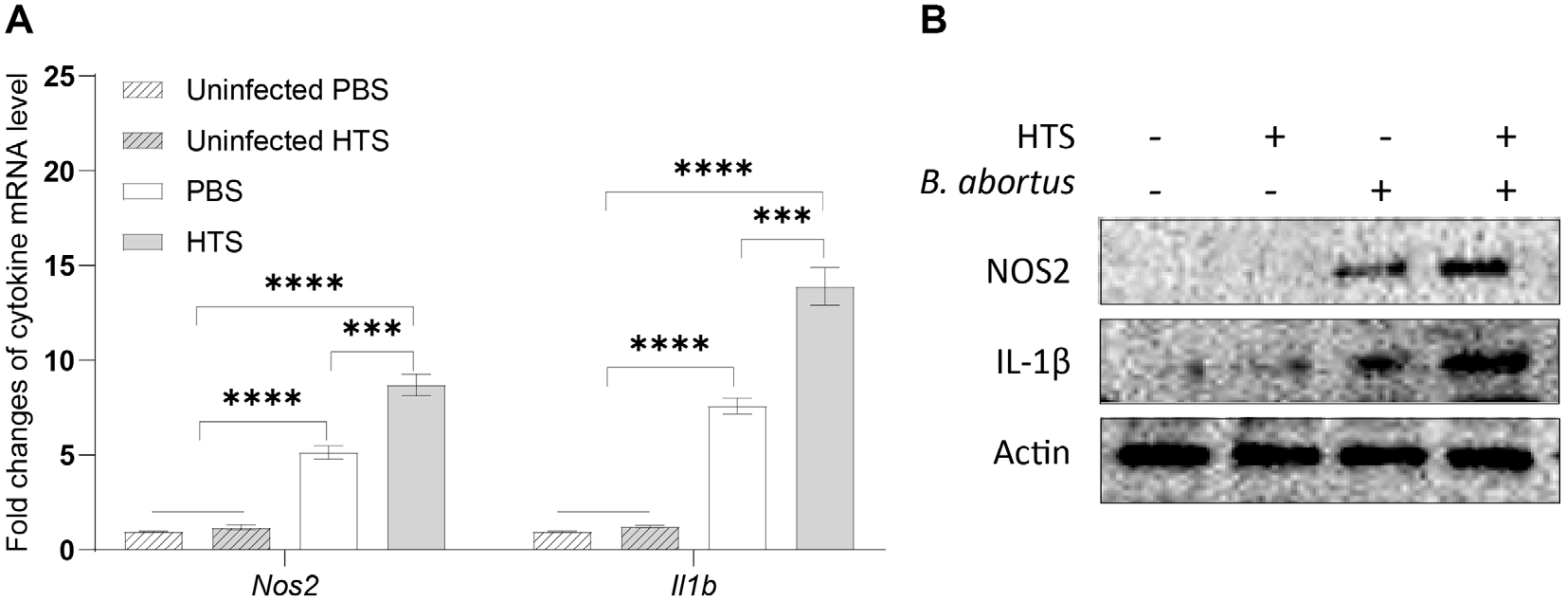
HTS treatment effect on the expression of IL-1β and iNOS in splenic cells. The transcriptional and translational expression levels of IL-1β and iNOS from splenic cells were quantified by qRT-PCR (**A**) and western blotting analysis (**B**), respectively. Asterisks indicate significant differences (***, *p* < 0.001; ****, *p* < 0.0001).

It is well-known that the expression of IL-1β and NOS2 induces host inflammatory responses. In this study, the expression of these markers in splenic cells were evaluated by qRT-PCR and western blotting assay. The transcription of both *Il1b* and *Nos2* genes was similar in uninfected groups with and without HTS treatment, however, *Brucella* infection induced the expression of these markers and concurrent HTS treatment significantly boosted this induction (Fig. 2A). Likewise, we were unable to detect IL-1β and NOS2 in uninfected cells, however, *Brucella* infection markedly induced the expression of these proteins, and concurrent HTS treatment elevated this induction (Fig. 2B). These findings demonstrated that HTS treatment significantly activated host inflammation upon *Brucella* infection.

### Effect of HTS on T helper 1 (Th1) and T helper 2 (Th2) T Lymphocytes Responses

**Fig. 3:**
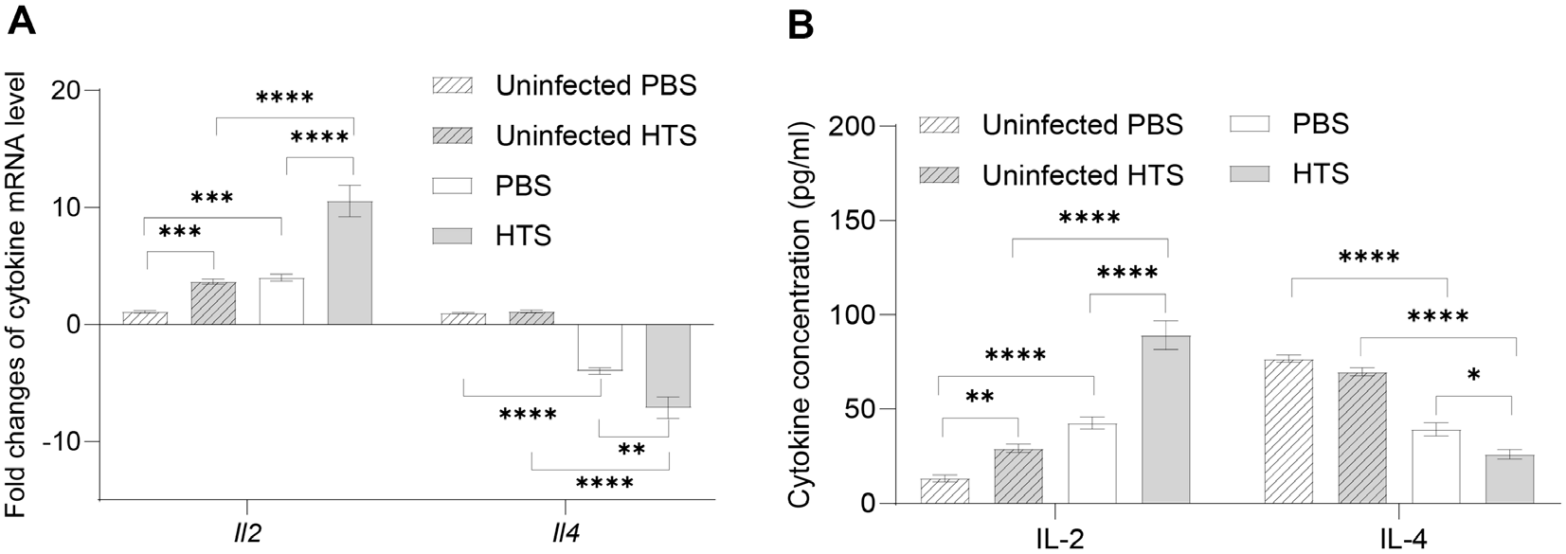
The effect of HTS on the production of Th1 and Th2 cytokines. Expression of cytokines IL-2 and IL-4 in splenic cells with and without exposure to HTS quantified by qRT-PCR (A) and ELISA (B). The data are represented as the mean ± SD of the mean of each group of six samples. Asterisks indicate statistically significant differences (*, *p* < 0.05; **, *p* < 0.01; ***, *p* < 0.001; ****, *p* < 0.0001).

Cytokines IL-2 and IL-4 play a dominant role in directing the differentiation of Th0 into Th1 and Th2 T cells, respectively [18]. Therefore, we examined the relevance of IL-2 and IL-4 expression in splenic cells and sera under HTS exposure during *Brucella* infection. Interestingly, the results proven by qRT-PCR and ELISA showed the same pattern of IL-2 and IL-4 production. Particularly, the IL-2 production from HTS-treated groups was significantly higher than those of untreated groups in both non-infected and infected groups. Notably, a substantial reduction of IL-4 production was observed from the spleens and sera of HTS-treated mice compared to untreated mice upon *Brucella* infection. These results indicated that the HTS probably directed the T lymphocytes to differentiate into Th1, an essential T cell subset for protection against *Brucella* infection (Fig. 3).

### HTS Treatment Regulated the Signaling Pathways of MAPKs, Autophagy and NF-ĸB

**Fig. 4:**
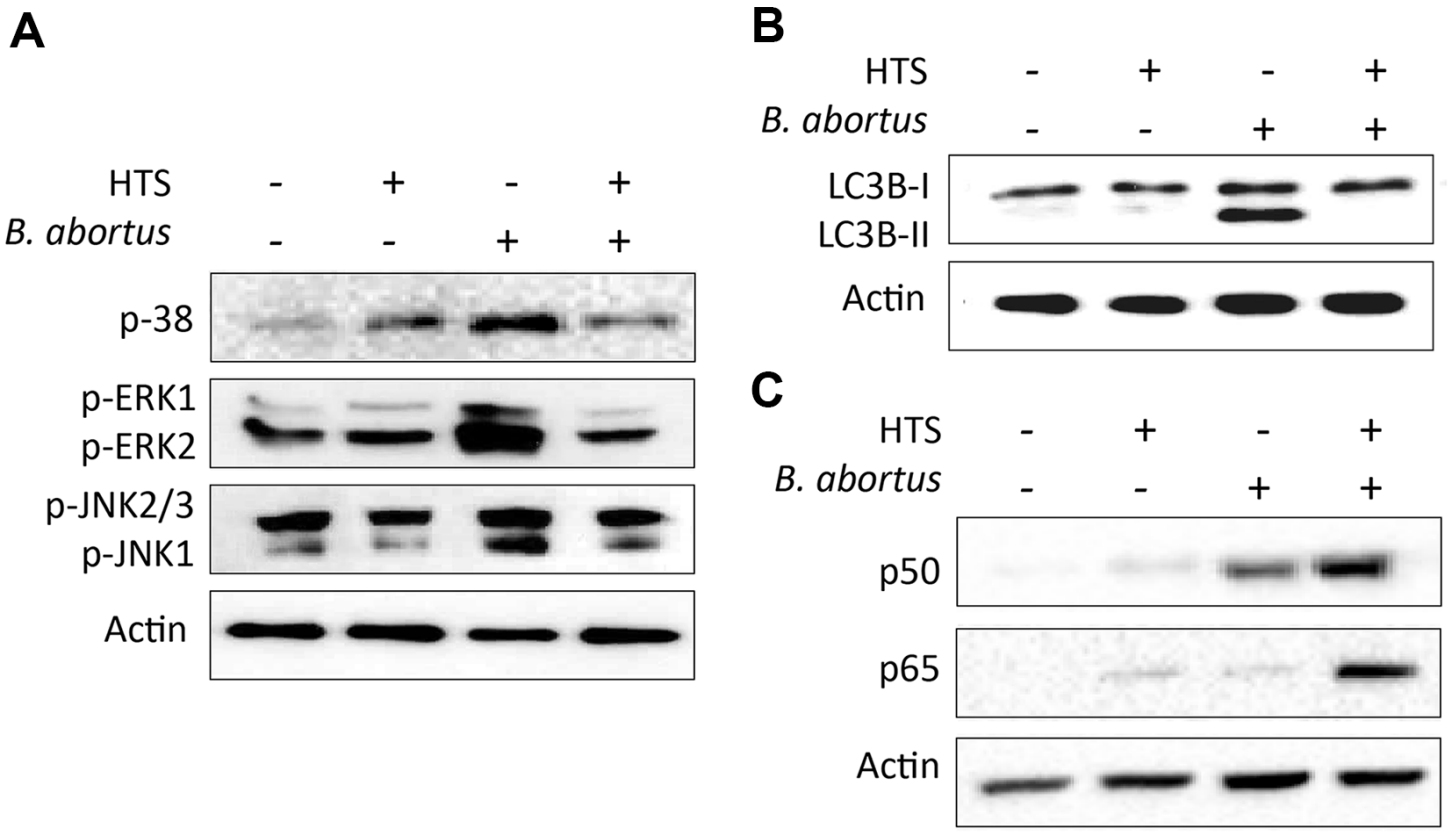
HTS regulates the expression of signaling pathways related to MAPKs (A), autophagy (B) and transcription factor NF-ĸB in splenic cells (C). Total proteins were extracted from mouse spleens by RIPA lysis buffer. The protein samples were subjected to western blotting assay with β-actin used as reference protein.

In order to understand the activation of signaling pathways contributing to host immunity under HTS treatment. The expression of MAPK signaling cascade, autophagy markers LC3B-I and LCB-II, and transcription factor NF-ĸB in splenic cells was investigated using western blotting assay. The results showed that the phosphorylation levels of p38, ERK1/2 and JNK were reduced in HTS-treated groups compared to untreated group during *Brucella* infection. Moreover, in the non-infected groups, HTS treatment slightly increased the expression of p38 (Fig. 4A). On the other hand, in the infected groups, an apparent down-regulation of the lipidated form LC3B-II was observed under HTS treatment (Fig. 4B). NF-ĸB is well-known as a key effector in regulating host immunity and inflammatory responses and we interestingly found that the expression of the members of NF-ĸB family, p50 and p65, were upregulated in splenocytes isolated from the HTS-treated infected group compared with the untreated infected group (Fig. 4C). These findings supported the notion that HTS treatment has effects on regulating signaling pathways that favor host immunity against *Brucella* infection.

### Protective Efficacy of the HTS Treatment in BALB/c Mice against *Brucella* Infection

**Fig. 5:**
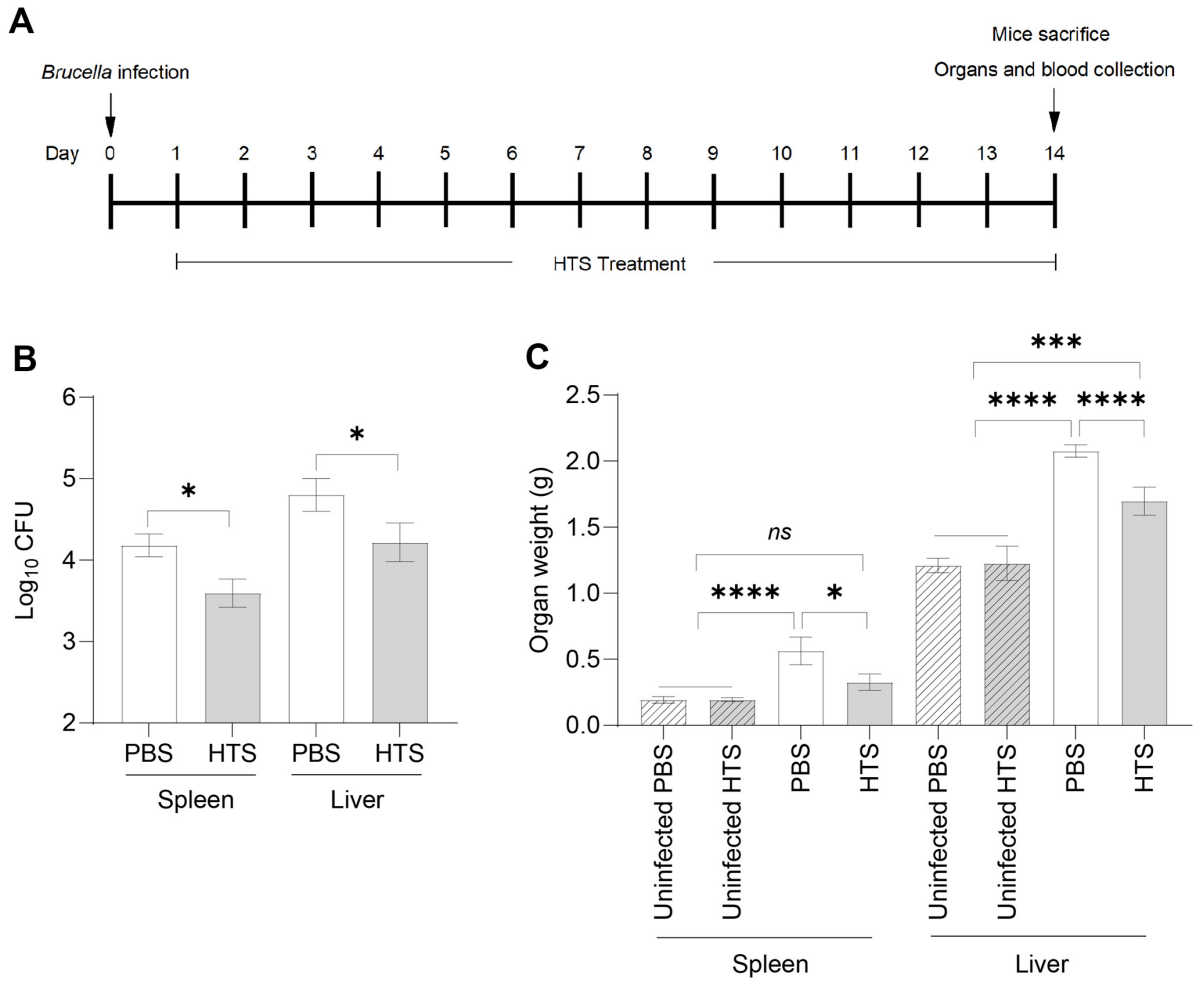
Protection against *Brucella* in BALB/c mice treated with HTS. BALB/c mice were intraperitoneally challenged with *B. abortus*, followed by daily oral treatment with HTS for 14 days. On the 14^th^ day post-infection, spleen and liver samples were collected (**A**) The bacterial burden in spleen and liver were evaluated and calculated with a base- 10 logarithm of the number of CFU (**B**). At the same time, spleen and liver weights (**C**) were also measured. The data are represented as the mean ± SD of the mean of each group of six samples. Asterisks indicate statistically significant differences (*, *p* < 0.05; ***, *p* < 0.001; ****, *p* < 0.0001).

After obtaining potential results related to the immunomodulatory effects of HTS, we elucidated the protective efficacy through the rate of bacterial clearance (log_10_CFU) together with splenomegaly and hepatomegaly during *Brucella* systemic infection. In uninfected groups, the total weight of the spleens and livers in HTS-treated groups displayed no difference compared to that of the control groups. Whereas in the case of *Brucella* challenge, HTS treatment in mice displayed significantly reduced splenomegaly and hepatomegaly compared to the untreated groups. Interestingly, a modest reduction of bacterial load was observed from the spleen of HTS-treated mice (3.65 ± 0.15) compared to control mice (4.18 ± 0.15). The same pattern of protective effect was also found in the liver organ. The results showed that HTS-treated groups (4.23 ± 0.26) exhibited a lower bacterial burden in the liver compared to control mice (4.82 ± 0.17). Taken together, we found that HTS treatment activated host immunity to combat against *Brucella* systemic infection, accompanied by attenuation of splenomegaly and hepatomegaly (Fig. 5, Table 2).

## Discussion

*Brucella* species are intracellular pathogenic bacteria that employ stealthy strategies to manipulate host immune systems. These strategies include hindering innate immune recognition, inhibiting phagolysosome fusion, impairing antigen presentation and adaptive immunity initiation, leading to the establishment of chronic infection [19]. At the onset of natural infection, *Brucella* first contacts the host body through the oral cavity then enters the mucous membranes of the gastrointestinal and respiratory tracts, the site for the establishment of *Brucella* infection [20, 21]. The innate immune system, as the first line of defense against pathogens, can be quickly activated after exposure to bacteria. This rapid response serves to inhibit bacterial replication, reduce the likelihood of systemic infection, and eliminate the infected bacteria at the onset of infection. Among meditators of innate immunity, the secretion of cytokines and chemokines plays an essential role in orchestrating other effectors of innate immunity, and even influencing adaptive immune response, to effectively combat bacterial infection [22]. A conspicuous pathogen-associated molecular pattern of *Brucella* is lipopolysaccharide, which is recognized by the TLR4-MD2 receptor-coreceptor complex. This binding mediates the production of several cytokines such as TNF-α, IL-12, IL-6, IL-1β, type I interferons, and IL-10 [23, 24]. Indeed, in this study, the stimulation of *Brucella* infection led to the secretion of cytokines IFN-γ, TNF-α, IL-6, IL-10 and chemokine MCP-1. IFN-γ activates macrophages to produce optimal NO and inhibit *Brucella* growth. Pro-inflammatory cytokines IL-6 and TNF-α were proven to promote phagolysosome fusion and production of reactive oxygen species, NO. Whereas anti-inflammatory cytokine IL-10 appears to suppress the production of IFN-γ, rendering the host susceptible to *Brucella* infection [25-27]. Similar to IL-10, the novel anti-inflammatory cytokine IL-1 family including IL-37 and IL-38 were involved in the regulation of chronic brucellosis [28]. Intriguingly, a growing amount of evidence is accumulating to support the regulatory effects of a high salt level in host pro-inflammatory responses and antimicrobial functions. A study by Chen *et al*. [14] demonstrated that HTS enhanced bacterial clearance and phagocytic activity by increasing Toll-like receptors (TLR) of inflammatory cells. Moreover, previous studies have shown enhanced systemic inflammatory responses through inflammasome activation and pro-inflammatory cytokine/chemokine production including IL-6, IL-1β, TNF-α and MCP-1, in response to high salt level exposure [12, 29, 30]. In this study, we found a significant elevation in the production of pro-inflammatory cytokines TNF-α, IL-6, IFN-γ and chemokine MCP-1 in the sera of mice exposed to HTS. Additionally, the overproduction of IL-10 in sera found in this study under HTS treatment was consistent with that proven in the previous study [31]. We suggest that the reduction of splenomegaly and hepatomegaly shown in this study were possibly affected by this significant enhancement of IL-10. Indeed, the lack of IL-10 caused the initial inflammation and pathology in the spleen and liver of Brucella-infected mice [32]. The induction of inflammatory responses is influenced not only by pro-inflammatory cytokine/chemokines but also by another potent marker of inflammation such as NO. And the effect of HTS on the expression of NO was clearly demonstrated in the present study and reported elsewhere [33]. These results underscore the critical role of HTS in stimulating host innate immune responses by enhancing inflammation.

A shift from a non-specific response against Brucella, innate immunity, to adaptive immune responses activated with an antigen-specific response and acquired immunologic memory. To eradicate *Brucella* infection at the late stage of infection, adaptive immunity triggers three major responses including activation of brucellacidal activities in macrophages by IFN-γ stimulation, killing of Brucella-infected immune cells by cytotoxic T lymphocytes – a main source of IFN-γ secretion, and opsonization of the pathogen by Th1- type antibody isotypes IgG2a and IgG3 [34, 35]. Th1 and Th2 T lymphocyte subsets are critical regulators affecting the outcome of brucellosis. Herein, cytokines play an important role as a biological rhythm in the priming of Th1 and Th2 activation. IFN-γ and IL-2 production is marker for Th1 priming, while IL-10 and IL-4 production closely relate to Th2. Among them, Th1 cells are well-known as a key effector in the clearance of *Brucella* infection [24]. In this study, HTS treatment showed increased expression of IL-2 while reducing the Th2 cytokine IL-4. In addition, although our results showed a significant elevation of IL-10 production in sera under HTS treatment, the production of IFN-γ was much higher than IL-10. Moreover, cytokine IFN-γ mainly is produced by CD4^+^ and CD8^+^ T cells and these T cell populations play essential role in Th1 immunity [36, 37]. Therefore, these findings suggest that Th1 immune response was predominant in *Brucella* infection during HTS treatment.

Autophagy, a cellular degradation mechanism, is capable of the removal of aged, dysfunctional organelles and the elimination of intracellular microbes. Another of Brucella's stealthy mechanism is the selective subversion of autophagy to favor its intracellular survival. Autophagosomes are activated after *Brucella* infection, as indicated by an increase in the autophagy marker protein LC3B-II, followed by *Brucella* replication in its preferred niche, endoplasmic reticulum [38, 39]. Therefore, the expression of LC3B-II under HTS exposure was examined in the present study. As a result, HTS treatment inhibited the expression of LC3B-II during *Brucella* infection. Previously, the reduction of LC3B-II expression caused by high salt treatment was demonstrated in the context of vasculoprotection [40, 41]. The MAPK signaling cascade was found to regulate the autophagy process. The induction of autophagy by salinomycin, a potent anticancer drug, was decreased by treatment of MAPKs p38 and ERK1/2 signaling cascade inhibitors in prostate cancer cells [42, 43]. Interestingly, in this study, HTS clearly dampened ERK1/2 and p38 activity. This is similar to the previous article, which indicated a negative regulatory effect of HTS on the phosphorylation of p38 and ERK1/2 through PKA signal transduction pathway in a human neutrophil model [44]. These results suggested that HTS treatment dampened the autophagy process by inactivating the signaling pathway MAPKs.

The transcriptional factor NF-ĸB is generally known as a key player in regulating host immune responses. The activation and nuclear translocation of NF-ĸB trigger the transcription of various genes related to innate immunity, which is associated with an inflammatory response for resistance to *Brucella* infection [45]. A previous study reported the implication of NF-ĸB in the expression of glutaminase gene results in M1 macrophage polarization and release of pro-inflammatory factors TNF-α, NOS, and IL-1β, contributing to *Brucella* clearance [46]. Moreover, NF-ĸB cascade is a significant contributor to the differentiation of Th1 by activating the production of Th1 cytokines. In this study, an increase in the production of cytokines associated with inflammation and Th1 development was demonstrated under HTS treatment, accompanied by a significant increase in NF-ĸB p50 and p65. Interestingly, our results align with a previous study that reported an increase in the phosphorylation of NF-ĸB and the production of IL-6 and MCP-1 due to high salt [47, 48]. On the other side, having obtained potential results in host immune response that favors *Brucella* clearance, we finally dissected the protective efficacy of HTS administration against *Brucella* persistence in the spleen and liver. A protective effect was conferred by HTS treatment against *Brucella* infection in a mouse model. This result is congruent with a previous study which showed that the HTS treatment significantly dampened the bacterial load in blood, lung, and spleen when mice were subjected to sepsis caused by *E. coli* *in-vivo* while increasing the intracellular killing and superoxide generation in neutrophils against *E. coli* infection *in-vitro* [13]. These findings suggest a critical role of HTS in augmenting host immune responses, thereby limiting *Brucella* systemic infection.

## Conclusion

In conclusion, this study provides further evidence supporting the beneficial effects of HTS treatment in controlling bacterial infection. HTS positively regulates host immune responses, favoring host resistance and promoting brucellacidal activity. In addition to its critical roles in innate immunity through the activation of the pro-inflammatory cascade, HTS also induces the secretion of Th1 cytokine markers, advancing the host’s killing mechanism to fight off *Brucella* infection. Both arms of the host immunity were proven to be regulated by the activation of NF-ĸB transcription factor. Another mechanism involved in *Brucella* resistance within innate immune system is autophagy, which was also down-regulated under HTS treatment through the MAPK signaling pathway. Although this study provided a preliminary understanding of the regulation of innate and adaptive immunity, further investigation into in vitro molecular mechanisms should be conducted to have a more comprehensive insight into the immunomodulatory effects of HTS in the context of *Brucella* infection.

## Figures and Tables

**Table 1:** Primer sequences used for qRT-PCR.

Gene	Common name	Forward primer	Reverse primer
*Il2*	Interleukin 2	5’- CTGGGGAGTTTCAGGTTCCTG -3’	5’- CTCGCATCCTGTGTCACATTG -3’
*Il4*	Interleukin 4	5’- GTCATCCTGCTCTTCTTTCTC -3’	5’- CACCTTGGAAGCCCTACAGAC -3’
*Il1b*	Interleukin 1β	5’- -CAACCACACAAGTGATATTC-3’	5’- GGATCCACACTCTCCAGCTG-3’
*Nos2*	Nitric oxide synthase 2	5’- GGAGGTGCTTGAAGAGTTCC -3’	5’- AGGAGGTGATGGAGTAGTAGC -3’
*b-actin*	β-actin	5’- CGCCACCAGTTCGCCATGGA-3’	5’- TACAGCCCGGGGAGCATCGT-3’

**Table 2:** Protection against *B. abortus* in mice treated with HTS.

Tissues	Treatment	Log_10_ CFU of bacteria (Mean ± SD)	Log protection	*P*-value^a^
Spleen	PBS	4.18 ± 0.15	0.53	*P* < 0.05
	HTS	3.65 ± 0.15		
Liver	PBS	4.82 ± 0.17	0.59	*P* < 0.05
	HTS	4.23 ± 0.26		

^a^Significantly different from PBS-treated mice were estimated by Student’s *t*-test.
